# Regulation of Clinical Research Sponsored by Pharmaceutical Companies: A Proposal

**DOI:** 10.1371/journal.pmed.0030306

**Published:** 2006-07-11

**Authors:** Julio Sotelo

## Abstract

Sotelo proposes that a third party, appointed by academic institutions, must be compulsorily placed between clinical researchers and drug companies.

Drug development is indispensable to deal effectively with disease [ 1]. However, two dilemmas emerge from clinical research devoted to this goal. First, new drugs are the source of a huge economy ruled not by the standards of the medical profession, but by the rules of business and the market [ 2]. Second, although the testing and the medical community's “blessing” of a new drug lie entirely in the hands of medical institutions, medical journals, and regulatory agencies [ 3], once the drug is approved the ruthless mechanisms of industry propaganda and commerce take over [ 4, 5].

The gains in health obtained by the advent of a new drug should always be considered according to the crucial concept of its cost–benefit balance, and the benefits often fall far below the costs (either monetary or otherwise). Of course, patients and drug companies might have different feelings about the costs and benefits of a new drug: patients have high expectations that the drug will help them to recover their health, whereas companies rely mostly on statistical arguments to back up the superiority of their new drug over existing ones [ 2, 3].

## Academic Medicine and the Pharmaceutical Industry: An Uneasy Relationship

The marriage between pharmaceutical companies and academic medicine at times becomes a love–hate relationship [ 4]. Nonetheless, it is an indissoluble link that has brought countless benefits to society [ 1], that is bound to continue, and that must surely be supported. But the medical profession, not the pharmaceutical companies, must find a way to base the relationship upon the academic and ethical aims that guide the practice of medicine and the public interest. [Fig pmed-0030306-g001]

**Figure pmed-0030306-g001:**
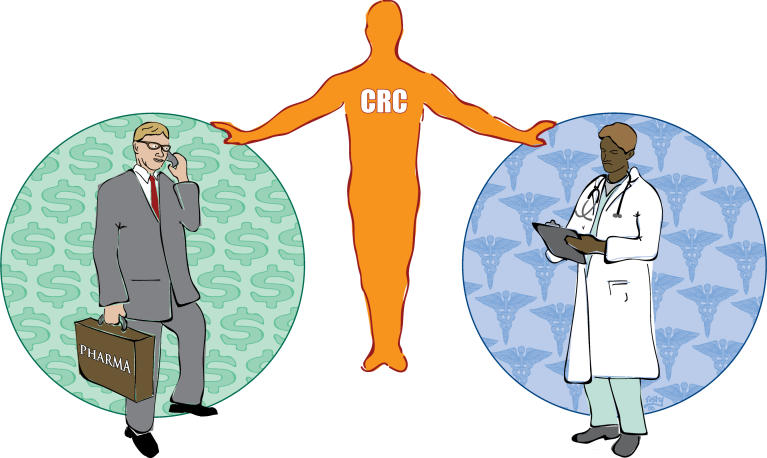
A third party—the CRC—should be placed between drug companies and clinical researchers (Illustration: Rusty Howson, sososo design)

Over the last few years, one scandal after another has shown how drug company marketing can distort prescribing patterns. The root of the problem seems to be simple: medical research sponsored by drug companies is a mixture of, on the one hand, impeccable scientific talent devoted to the well-being of society, and, on the other, marketing expectations on the side of the company sponsoring the research. Both actors are effective professionals, but their aims could not be more distant. In some instances sponsors control the trial design, interpretation of results, writing up of study results, and publication strategies.

A number of proposals have been put forward to reduce the influence of the pharmaceutical industry upon medical practice. These include the creation of an independent institution to oversee clinical testing of prescription drugs [ 5], strict regulation of drug marketing [ 6], and rigorous guidelines for publication of pharmaceutical research in scientific journals [ 3].

My proposal to realign the relationship between clinicians and drug companies is simple and is based on a single premise. I propose that a third party, appointed by academic institutions, must be compulsorily placed between clinical researchers and drug companies so that all dialogue (scientific and monetary) must be made through this third party. The guarantors of this new proposal would be the scientific journals, where the results of the research are intended to be published [ 7].

## A Proposal for a Collegiate Research Council

I propose that a committee called a Collegiate Research Council (CRC) be jointly appointed by 10–12 leading academic institutions. This committee would be certified by the appropriate health authorities (such as the United States Food and Drug Administration or equivalent agencies in other countries).

The CRC would be the only recipient of a research protocol related to the clinical testing of a drug; the pharmaceutical company or contract research organization must submit the protocol to the CRC. The CRC would define the costs of the project, including the salaries of the scientific staff involved and appropriate compensation for the institutional review boards and for the CRC [ 8]. The CRC would select the optimal investigators and institutions based on their expertise, prestige, and independence, and would offer them, without the participation of the pharmaceutical company, the opportunity to conduct the trial. In this way, the “sponsor” of the trial would be the CRC, not the pharmaceutical company.

The local institutional review boards (or ethics committees) would review and approve the trial protocol, just as they currently review protocols [ 8, 9]. The investigators selected must be independent from both the CRC and the pharmaceutical company. When it came to financial matters, the clinical researchers would deal only with the administrators appointed by the institution or the CRC (the researchers would not deal directly with the drug companies).

The CRC would monitor the randomization process; it could choose to hire autonomous experts to review the design of the trial. The results of the trial would be analyzed by the CRC prior to publication. These results would be made public, even if they were negative. The results of the trial would be written by the investigators with total independence and submitted for publication to an interested journal, acknowledging that the research was conducted according to the guidelines of the CRC.

## Benefits of the Proposal

I believe that this regulatory proposal would prevent several biases and would improve the reliability of clinical studies, which would obviously be in the public interest. The natural reticence of the pharmaceutical industry to embrace this regulation would lessen with time, in my opinion, because the scholarly support for the reliability of any given trial would be greatly enhanced. In this way, it would be expected that accusations and lawsuits against drug companies would diminish because of the prestige of the collegiate boards that participate in the whole process of testing a drug before its eventual approval for marketing. This procedure would also enhance public confidence in the scientific basis of novel drug treatments.

Currently, the academic boards of any given institution review the ethical and methodological aspects of a research proposal. As expected, the protocols sponsored by pharmaceutical companies are usually adequate, as they are designed by experts hired by the industry [ 3]; therefore, the protocol is commonly approved by the respective boards. The problem does not lie in the design of trials, rather, in the very fact that the drug company pays the researcher directly. This payment changes the relationship from that of scientist and businessman to that of employer and employee, with all the characteristics and inequalities that this relationship entails. Currently, the investigator receives fees, grants, travel expenses, and other gifts from the company that owns the drug that is under investigation, generating in the investigator natural feelings of gratitude towards the generosity of the sponsor. The relationship creates unfavorable grounds for proper scientific evaluation, because it creates potential bias in the analysis of the trial results. The compulsory presence of the CRC would break this bond by introducing in the middle a reliable, well-meaning, highly respected and vigilant party between the drug company on one side and the investigator on the other side.

The proposed CRC would also prevent the drug company from having the power to decide whether or not the results of a trial will be published. Currently, many trials go unpublished (particularly negative trials) [ 10]. Patients must surely be informed about all clinical research, including negative results, relevant to a drug that they are going to take.

Although difficult to achieve, I also propose that the CRC should be informed about the intended price for the drug. This would allow the CRC to have a say on the crucial subject of the cost–benefit balance, which nowadays is left solely in the hands of the drug manufacturers. I believe that the medical profession, which is accountable to society (not just to individual patients), should be more actively involved in monitoring the way in which drugs become commercialized—particularly given that it is clinical researchers and clinicians who will test and then prescribe them. The intervention of the medical profession in discussions of costs and benefits would likely have strong public support, particularly given that many new treatments are discovered through publicly funded research. It seems unfair that the price of a drug is currently set arbitrarily by the drug manufacturer, without the involvement of the medical community. Also, in contrast with the commercial trade of other goods, where prices are set by production costs and competition, in the peculiar case of pharmaceutical substances the price is not related to production costs or competition. Instead, drug companies justify the high prices of drugs on the weak argument of high “research costs,” an argument which has been so eloquently disputed by Marcia Angell in her book *The Truth about Drug Companies: How They Deceive Us and What to Do About It [ 5].*

## Conclusion

Although this proposal is brief, it could serve as a draft for devising a more comprehensive mechanism of regulation headed by a CRC. The participation of the CRC would not represent a large economical investment nor an additional bureaucratic body to delay the testing of new drugs. Instead, I believe that it would lower the current spending by pharmaceutical companies on clinical trials and would accelerate the process of drug testing and drug approval on behalf of the patient.

## Abbreviation

CRC: Collegiate Research Council
